# Development of a Machine Learning-Based Prediction Model to Differentiate Infectious and Non-Infectious Diseases in Patients with Undiagnosed Fever: A Single Hospital-Based Retrospective Study

**DOI:** 10.3390/jcm15051905

**Published:** 2026-03-02

**Authors:** Masahiko Nakamura, Shun Yamashita, Ryosuke Osako, So Motomura, Naoko E. Katsuki, Shu-ichi Yamashita, Masaki Tago

**Affiliations:** 1 Department of General Medicine, Saga University Hospital, Saga 849-8501, Japan; masahikonakamura39@gmail.com (M.N.); somotomurabhvz@gmail.com (S.M.); d4180@cc.saga-u.ac.jp (N.E.K.); tagomas@cc.saga-u.ac.jp (M.T.); 2 Department of Emergency and Critical Care Medicine, University of Miyazaki Hospital, Miyazaki 889-1692, Japan; 3 Education and Research Center for Community Medicine, Faculty of Medicine, Saga University, Saga 849-8501, Japan; 4 Department of Cardiovascular Medicine, Saga University, Saga 849-8501, Japan; ryosuke.osako1@gmail.com; 5 Department of Internal Medicine, Heiwadai Hospital, Miyazaki 880-0034, Japan; syama@cc.saga-u.ac.jp

**Keywords:** prediction model, machine learning, serum ferritin level, white blood cell count, neutrophil percentage, platelet count, lactate dehydrogenase level, infection, undiagnosed fever

## Abstract

**Background/Objectives**: Fever can develop from several causes, including infectious diseases, noninfectious inflammatory diseases (NIID), malignancies, and other medical conditions. Although serum ferritin (SF) level can help differentiate infectious from non-infectious diseases, its discriminative ability (specificity) is far from satisfactory. The aim of this study was to develop a diagnostic prediction model to distinguish infectious diseases from other febrile illnesses using only common blood tests available on admission, in addition to SF level, in patients with undiagnosed fever. **Methods**: This single-center retrospective observational study included patients with fever of unidentified origin aged ≥18 years admitted to a Japanese acute care hospital between 1 January 2013, and 31 December 2022. They were divided into infectious and non-infectious disease groups based on their final diagnosis. Machine learning and multivariable logistic regression analysis were used to develop a model to differentiate infectious diseases from non-infectious diseases. Model performance was evaluated using area under the curve (AUC), shrinkage coefficient, and stratified likelihood ratio. **Results**: Among the 143 patients included, 73 had infectious diseases. A prediction model consisting of five factors—serum white blood cell count, neutrophil percentage, platelet count, lactate dehydrogenase level, and log-transformed SF level—was developed. The AUC of the model was 0.794 (95% confidence interval: 0.721–0.867) with a sensitivity of 77.1%, specificity of 68.5%, shrinkage coefficient of 0.876, and stratified likelihood ratio of 0.13–5.04. **Conclusions**: We developed a prediction model consisting of only five high-performing indicators, which would help differentiate infectious diseases from other fever causes early after admission.

## 1. Introduction

Fever is one of the most common complaints in clinical practice and can be caused by infectious diseases, noninfectious inflammatory diseases (NIID), malignancies, and other medical conditions [1,2]. Identifying and correctly diagnosing the cause of fever remains elusive. Recently, it has been reported that 15–20% of cases of classical unidentified fever remain undiagnosed, even with the existence of highly developed diagnostic equipment and techniques [2,3,4,5], possibly leading to unnecessary administration of antibiotics or steroids [6]. This can cause various adverse events, including the induction of drug resistance in bacteria [6,7] or drug-induced organ damage, such as pseudomembranous enteritis in patients [8,9], and steroid use for infectious diseases could exacerbate damage from the disease by weakening the immune system [10]. Therefore, differentiating between infectious and non-infectious diseases early after admission may help reduce unnecessary use of antibiotics or steroids.

Serum ferritin (SF) level has been reported to be useful in inferring causative diseases of fever [11,12]. Furthermore, SF level on admission has been reported to be effective in differentiating between infectious and noninfectious diseases, with an area under the curve (AUC) of 0.75 for the former [11]. However, another study showed a lower AUC of 0.64, suggesting that SF level alone may be insufficient for this purpose [6]. Accordingly, several models have been developed to distinguish between infectious and non-infectious diseases as causes of unknown fever. Although one such model showed a high AUC of 0.79–0.80, it requires the collection of several parameters in a short timeframe, including sex, presence or absence of chills or shivering, presence or absence of lymphadenopathy, presence or absence of pleural effusion, presence or absence of hepatosplenomegaly, serum lactate dehydrogenase level (LDH), SF level, serum procalcitonin level, antinuclear antibody, or mycobacterium tuberculosis interferon gamma [13]. Another model exhibited a high AUC of 0.775 [6]. However, it requires serum amyloid protein level, which is rarely examined, to identify the cause of fever of unknown origin, making it impractical for use in daily clinical practice. Therefore, it is desirable to develop a simple and easy-to-use prediction model involving readily available factors.

The aim of this study was to develop an easy-to-use prediction model for differentiating between infectious and non-infectious diseases using only blood tests commonly measured on admission, with SF level among patients aged 18 years and older who were admitted with undiagnosed fever.

## 2. Materials and Methods

### 2.1. Study Design and Participants

This single-center retrospective observational study included patients aged 18 years and older who were admitted to Saga University Hospital between 1 January 2013, and 31 December 2022, with undiagnosed fever. Patients assigned the International Statistical Classification of Diseases and Related Health Problems—10th Revision (ICD-10) code of R-50-9 for fever of unknown origin during admission were identified from medical records. Patients without measurement of SF level from the day before to three days after admission, without fever over 37 °C before admission, and with a definitive diagnosis of the cause of fever before admission were excluded. In addition, patients who failed to undergo adequate outpatient investigations of the cause of fever before admission, including chest X-ray, blood tests, and urinalysis (including urine culture), were excluded, even if alternative tests such as chest computed tomography (CT) were performed. In Japan, owing to its unique medical insurance system, it is not permitted to take a chest CT without performing chest X-ray. Therefore, in this study, chest CT did not serve as a substitute for chest X-rays. Finally, patients without a confirmed fever diagnosis were excluded from the analysis.

### 2.2. Setting

The present study was conducted at Saga University Hospital, the only university hospital in the Saga prefecture (population 811,000), a regional city in Japan. Saga University Hospital is a tertiary care hospital with 602 beds and 29 departments, including the Departments of General Medicine, Infection Control, and Collagen Diseases, which provide advanced medical services, mainly for acute patients.

### 2.3. Data Collection

Two physicians from the Department of General Medicine reviewed electronic medical records to extract patient data. The following information was extracted: age, sex, admission and discharge dates, length of hospital stay, ambulance transfer status, and mortality during hospitalization; execution rates of diagnostic tests including blood culture, sputum culture, cerebrospinal fluid culture, echocardiogram (separately tallied for transthoracic and transesophageal echocardiography), plain chest X-ray, cranial CT, chest CT with contrast enhancement, cranial magnetic resonance imaging (MRI), spinal MRI, upper esophagogastroduodenoscopy, colonoscopy, cytology, histopathological examination, interferon-gamma release assay, syphilis testing, and serum β-D-glucan level; and blood tests on admission including white blood cell count, neutrophil percentage, platelet count, and levels of C-reactive protein, D-dimer, fibrin/fibrinogen degradation products, LDH, albumin, total bilirubin, aspartate aminotransferase, alanine aminotransferase, blood urea nitrogen, creatinine, sodium, potassium, chromium, and ferritin. Administration of antibiotics administered before obtaining blood culture samples was also investigated, except in patients on long-term antibiotics for chronic bronchitis. Among the blood test parameters, serum white blood cell count, neutrophil percentage, platelet count, and levels of C-reactive protein, LDH, albumin, total bilirubin, blood urea nitrogen, and creatinine were surveyed because they have been reported to be useful in the diagnosis or prediction of the prognosis of infectious diseases [14,15,16]. Serum LDH level and neutrophil percentage have also been reported to be useful for differentiating infectious from non-contagious diseases in previous studies [6,13]. Other survey items were determined through discussions among members of the study group. The SF level measured on a day closest to the day of admission, within the period from the day before to 3 days after admission, was used, because there was a period during which constant measurement was not possible at the study institution. Administration of iron or transfusion of concentrated red blood cells within three months before admission, which could affect SF levels, was also surveyed. For the final diagnosis, after two physicians with over 10 years of post-graduation experience independently determined the diagnosis using the ICD-10 codes based on medical records, the diagnosis agreed upon by them was recorded as the final diagnosis. In the case of disagreement, the final diagnosis was determined by discussion involving both physicians and a third physician.

### 2.4. Statistical Analysis

Missing values were excluded from univariate and multivariable analyses. Among tests performed for diagnosis, unperformed tests were classified as “no tests.” Patients were divided into two groups: infectious disease group and noninfectious disease group. Patients with a final diagnosis of infectious disease were enrolled in the infectious disease group, whereas those with malignancies, NIID, or other diseases were enrolled in the non-infectious disease group. Categorical variables were expressed as percentages and compared using the χ^2^ test. Continuous variables were expressed as medians and quartiles and compared using the Mann–Whitney U test. Natural logarithmic transformation was applied to right-skewed continuous variables. Statistical significance was set at *p* < 0.05. To identify candidate variables for the model, we applied two approaches: the Boruta algorithm and Least Absolute Shrinkage and Selection Operator (LASSO) regression. Variables with missing values were also excluded from the candidate predictors for the model. To examine whether missingness in excluded variables influenced the final diagnostic grouping, we performed univariable analyses comparing the presence or absence of missing values with the final diagnostic category for each excluded variable. Multivariable logistic regression analysis was performed using candidate variables identified by each approach, and two models for differentiating infectious diseases from non-infectious diseases were developed. The predictive performances of the two models were compared using the Bayesian Information Criterion (BIC) value. Ultimately, the model with the lower BIC value was determined as the final model, and its sample overfitting was evaluated using the shrinkage coefficient. Stratum-specific likelihood ratios were also calculated. As a negative-control analysis, we generated scrambled data by randomly permuting the dependent variable while maintaining the predictor matrix unchanged; the identical logistic model was refitted, and performance was evaluated using the receiver operating characteristic AUC tested against 0.5. We calculated the probability of developing a non-infectious disease, sensitivity, specificity, positive predictive value (PPV), and negative predictive value (NPV) for each cut-off point corresponding to a sensitivity of 90%, Youden’s index of 0.80, or a specificity of 90% of the final prediction model. SPSS Statistics (version 25; IBM Corp., Armonk, NY, USA) and R software were used for statistical analyses. The Boruta package (version 8.0.0) and the caret R package (version 7.0-1) were used for selection of predictors and LASSO regression, respectively.

### 2.5. Sample Size

Based on an AUC of 0.775 for the existing model [6] and a null-hypothesis AUC of 0.5, we defined an effect size of 0.275. With α error of 0.05 and β error of 0.1, the required sample size was 16 patients in each group, resulting in a total of 32 patients. However, as a rule of thumb, ≥10 outcome events per predictor are required. Because we planned to construct a model consisting of three to five factors based on multivariable logistic regression analysis, the required sample size in the present study was 30 to 50 patients in each group, giving a total of 60 to 100 patients.

### 2.6. Ethical Considerations

This study conformed to the Ethical Guidelines for Medical and Health Research Involving Human Subjects in Japan. The study was conducted in accordance with the Declaration of Helsinki and approved by the Ethics Committee of Saga University Hospital (file number: 2023-11-R-07, approval date: 2 February 2024). Informed consent for participation was obtained from all subjects using an opt-out method through the homepage of the Clinical Research Center of Saga University Hospital. Patient anonymity was maintained.

## 3. Results

### 3.1. Enrollment and Allocation of Study Participants

**Figure 1:**
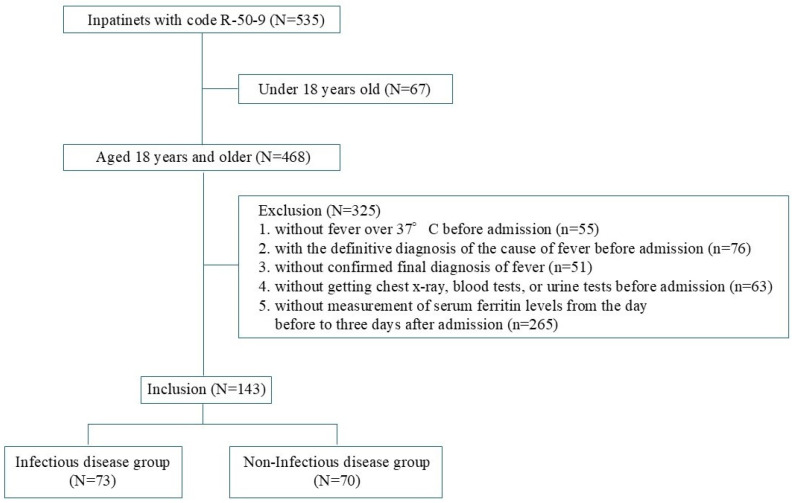
Enrollment and allocation of study participants.

Participant enrollment and allocation to the infectious or non-infectious disease groups are shown in Figure 1. Among the 535 patients with code R-50-9, 325 met the exclusion criteria and were excluded from the analysis. Of the remaining 143 patients, 73 and 70 were assigned to the infectious and non-infectious disease groups, respectively. Details of diseases included in both groups are shown in Supplement Table S1.

### 3.2. Patient Characteristics

As shown in Table 1, length of hospital stay was significantly longer in the non-infectious disease group than in the infectious disease group (12 days [interquartile range (IQR): 8–22] vs. 23 [13–38] days, *p* < 0.001). No differences were observed in age, sex, mortality, number of antibiotics administered prior to blood sample collection, ambulance transfer status, iron supplementation before admission, or patient outcomes between the two groups. The execution rates for these tests are presented in Supplement Table S2.

### 3.3. Laboratory Findings on Admission

As shown in Table 2, the following parameters were significantly lower in the infectious disease group than in the non-infectious disease group: platelet count (18.8 × 10^3^ [10.9 × 10^3^ − 26.5 × 10^3^]/µL vs. 27.6 × 10^3^ [15.0 × 10^3^ − 44.0 × 10^3^]/µL, *p* = 0.002), SF level (334 [191–713] ng/mL vs. 659 [361–1493] ng/mL, *p* < 0.001). Contrastingly, the following were significantly higher in the infectious disease group: blood urea nitrogen level (16.8 [11.4–24.9] mg/dL vs 13.3 [10.1–22.0] mg/dL, *p* = 0.044) and creatinine level (0.81 [0.64–1.17] mg/dL vs 0.73 [0.58–0.94] mg/dL, *p* = 0.048). Neutrophil percentage was not significantly different between the two groups (81.1% vs. 79.3%, *p* = 0.069). Full details on these laboratory tests in the groups are shown in Supplement Table S3.

### 3.4. Selection of Candidate Variables and Multivariable Logistic Regression Analysis

D-dimer, fibrin/fibrinogen degradation products, total bilirubin, serum iron, and TIBC were excluded from the candidate predictors because they contained missing values. None of these variables showed a significant association between missingness and the final diagnosis (all *p* > 0.05). The Boruta algorithm and LASSO regression identified five and thirteen candidate predictors, respectively (Supplement Figure S1). Using these identified predictors, two multivariable logistic regression analyses were performed (Table 3).

The BIC values of the Boruta-selected and LASSO-selected models were 187.646 and 205.456, respectively. Given the lower BIC level, the Boruta-selected model was determined as the final prediction model. A detailed prediction model formula is as follows:

−5.773 + 0.687 × ln [SF level (ng/mL)] + 0.059 × [platelet count (10^4^/µL)] − 0.001 × (neutrophil percentage) − 0.000014 × [white blood cell count (10^3^/µL)] + 0.000207 × [LDH (IU/L)]

**Figure 2:**
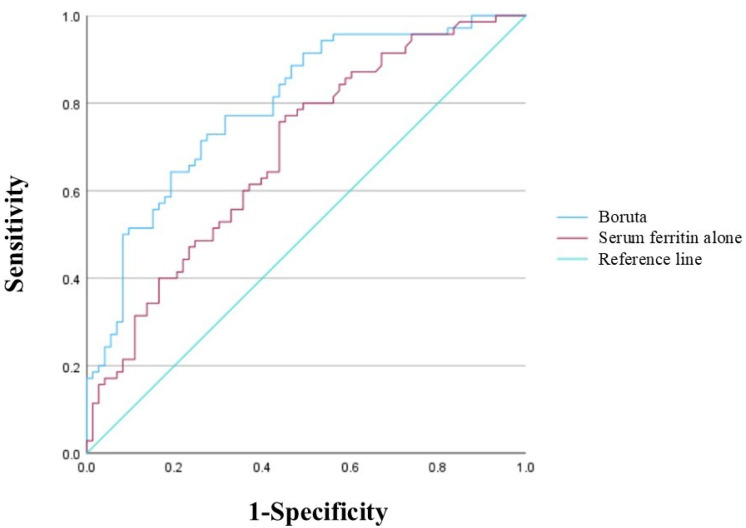
Receiver operating characteristic curve and area under the curve.

**Figure 3:**
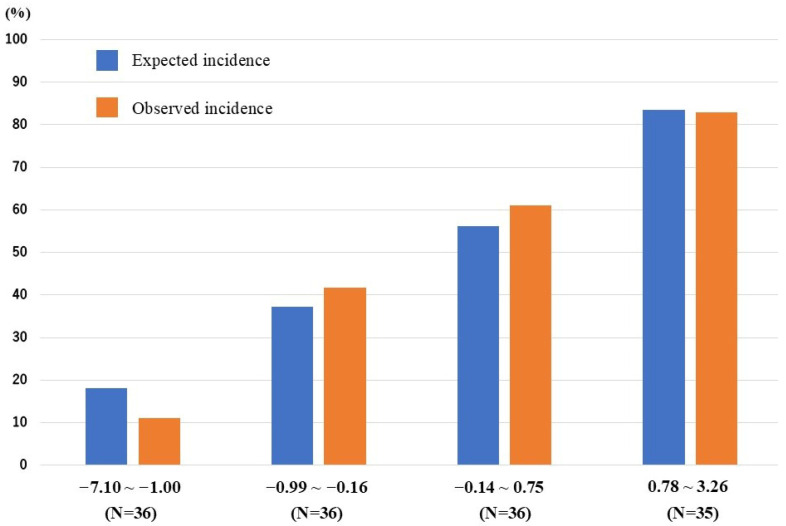
Predictive and observed rates of non-infectious disease in quadrisect groups according to scores in the internal validation cohort.

The AUC of the final model was 0.794 (95% confidence interval [CI]: 0.721–0.867) (Figure 2). The AUC for the SF level alone was 0.687 (95% CI: 0.601–0.773). On the scrambled data, the AUC was 0.569 (95% CI: 0.475–0.664, *p* = 0.152). The final model was well calibrated in the Hosmer–Lemeshow test (*p* = 0.356), with a shrinkage coefficient of 0.876 (Figure 3). The stratum-specific likelihood ratio was 0.13–5.04, increasing as the score increased (Table 4). The cut-off values of the predictive model scores for a sensitivity of 90%, Youden’s index, and a specificity of 90% were −0.85, −0.21, and 0.53, respectively. The PPV and NPV for each cut-off value were 36.4% and 84.1%, 71.9% and 69.6%, and 83.3% and 65.3%, respectively (Table 5).

## 4. Discussion

We developed a simple prediction model for differentiating between infectious and non-infectious diseases among patients with undiagnosed fever, consisting of log-transformed SF level, white blood cell count, neutrophil percentage, platelet count, and LDH. This model is easily available on admission to the majority of acute care hospitals in Japan. The AUC of our prediction model was 0.794 (95% CI: 0.721–0.867), showing good discrimination ability similar to that of existing complicated and cumbersome models that require many factors or rarely examined data in daily clinical practice, such as serum amyloid protein [6,13]. The five factors required by our model are simpler and easier to obtain, making it more usable and ideal for daily clinical practice compared with preexisting models.

Log-transformed SF levels may be particularly useful for differentiating between infectious and non-infectious diseases, because both were independent factors in a previous model [6]. Among diseases frequently associated with fever, hematological diseases and malignancies have been reported to show the highest SF levels, followed by NIIDs, with infectious diseases showing the lowest levels [4,11]. In the present study, SF level was also higher in the non-infectious disease group, in which patients with malignancy showed the highest values, followed by those with other diseases and NIID. Thus, SF level, a significant factor in the present study, would be useful in differentiating between infectious and non-infectious diseases.

White blood cell count and neutrophil percentage may also be significant factors. Neutrophil percentage is usually higher in bacterial infections than in infections caused by pathogens other than bacteria or non-infectious inflammatory diseases [17]. In addition, infectious diseases are a major cause in patients with fever of unknown origin, in which bacterial infections account for as high as 55–78% of cases [4,6]. In the present study, neutrophil percentage was a significant factor owing to its higher value in the infectious disease group, as approximately 70% of causative diseases in the infectious disease group were bacterial infections.

Platelet count was also detected as a significant factor in the present study, unlike in previous studies [6]. To the best of our knowledge, platelet count has never been reported as a useful marker for predicting infectious diseases, except in one report on sepsis in neonates [18]. Platelet count can markedly decrease in infectious diseases when progressing to sepsis or being complicated by disseminated intravascular coagulation [19], whereas it usually increases in autoimmune diseases, except when complicated by hemophagocytic syndrome, or in other exceptional subsets of autoimmune diseases [20]. For malignancies other than hematopoietic tumors, solid tumors tend to increase platelet count [21]. In the present study, platelet count was significantly higher in the non-infectious disease group, in which patients with NIID showed the highest platelet count, followed by those with other diseases and malignancies. The high proportion of hematopoietic tumors among malignancies (66%) may explain why patients with malignancies had the lowest platelet counts. In addition, the high proportion of NIIDs among non-infectious diseases (68%) may explain the higher platelet count in this group.

Finally, serum LDH was identified as a significant factor in the present as well as a previous study [13]. LDH is a commonly expressed enzyme across all organisms, especially in the heart, liver, muscles, lungs, kidneys, and blood cells [22,23]. Elevated serum LDH levels indicate tissue damage by non-infectious diseases, including solid cancers, autoimmune diseases, cardiovascular and cerebrovascular diseases, hematological disorders, and renal diseases [23]. Although the serum LDH levels are also increased in infectious diseases, such increases are usually associated with severe infections or a limited number of specific pathogens, including human immunodeficiency virus, coronavirus disease 2019, and malaria [22,23]. In our present study, the serum LDH level was higher in non-infectious disease group than in the infectious disease group. Because our study focused on patients admitted with undiagnosed fever, it included few such infectious diseases despite a substantial number of NIID and malignancies. Accordingly, serum LDH was selected as a predictor in the present study.

This study has some limitations. First, because it was a single-center retrospective observational study, selection bias may have occurred in the inclusion of participants and survey items. In addition, we were unable to compare serum ferritin levels before and after hospitalization because only a very small number of patients had follow-up measurements of serum ferritin after admission. The maximum serum ferritin level might represent useful information for distinguishing between infectious and non-infectious diseases. Second, 51 patients with fever of unknown origin as the final diagnosis were excluded from the analysis. In a previous report, approximately 12% of undiagnosed cases during hospitalization were eventually diagnosed more than 150 days after fever onset [24]. In our study population, approximately six patients were subsequently correctly diagnosed. However, they were excluded from analysis. If diagnoses were made during their hospitalization, the results of the present study may have been affected to some extent. Third, in this study, we did not investigate comorbidities or any history of unexplained fever. If these data had been collected, the patient profile for which this model should be applied might have been defined more specifically. Finally, the AUC of our prediction model was derived using the same cohort used to develop the model. To clarify whether our prediction model is applicable to various populations, it is necessary to perform a multicenter external validation study as the next step. Despite these limitations, our prediction model is more practical than all existing models for the differentiation of infectious from non-infectious diseases, because it includes only a few easily available, well-calibrated predictors with good discrimination ability.

## 5. Conclusions

We developed a simple prediction model for differentiating between infectious and non-infectious diseases in patients with undiagnosed fever on admission, consisting of the log-transformed SF level, white blood cell count, lactate dehydrogenase level, neutrophil percentage, and platelet count available on or around admission. Our model is useful owing to its good performance and simplicity. This model may help non-infectious disease specialists and general physicians differentiate infectious diseases from non-infectious diseases early after admission. Such early differentiation may lead to a reduction in inappropriate use of antibiotics or corticosteroids.

## Figures and Tables

**Table 1:** Univariate analysis of patient characteristics.

	Total (*n* = 143)	Infection (*n* = 73)	Non-Infection (*n* = 70)	*p* Value
Age, years	66 (51–79)	67 (49–80)	66 (52–78)	0.974
Male	71 (50)	36 (49)	35 (50)	1.000
Duration of hospital stay	16 (10–29)	12 (8–22)	23 (13–38)	<0.001
Mortality				
30-day mortality	7 (5)	3 (4)	4 (6)	0.657
In-hospital mortality	8 (6)	4 (6)	4 (6)	0.951
Transfer by ambulance	30 (21)	19 (26)	11 (15)	0.130
Administration of antibiotics ^※^	89 (62)	41 (56)	48 (69)	0.167
Administration of iron tablets	6 (4)	5 (7)	1 (1)	0.106
History of blood transfusions	7(5)	4(5)	3(4)	0.741
Outcome				0.426
In-hospital death	9 (6)	3 (4)	6 (9)	
Hospital transfer	33 (23)	19 (26)	14 (20)	
Discharge to home	101 (71)	51 (70)	50 (71)	

Categorical data are expressed as *n* (%) and were compared using the χ^2^ test. Continuous variables are expressed as median (interquartile range) and were compared using the Mann–Whitney U-test. ^※^ Prior to obtaining blood cultures.

**Table 2:** Univariate analysis of laboratory findings on admission.

	Total (*n* = 143)	Infection (*n* = 73)	Non-Infection (*n* = 70)	*p* Value
White blood cell count (×10^3^/µL)	9.6 (5.7–12.8)	8.8 (6.0–11.6)	10.1 (5.7–15.2)	0.152
Neutrophil percentage (%)	80.6 (72.8–86.2)	81.1 (75.8–87.2)	79.3 (66.4–85.5)	0.069
Platelet count (×10^4^/µL)	22.0 (12.3–36.2)	18.8 (10.9–26.5)	27.6 (15.0–44.0)	0.002
Albumin (g/dL)	2.8 (2.4–3.4)	3.0 (2.5–3.5)	2.8 (2.4–3.2)	0.070
Total bilirubin (mg/dL)	0.6 (0.5–0.9)	0.6 (0.5–0.9)	0.6 (0.4–0.9)	0.527
Aspartate aminotransferase (IU/L)	32.0 (21–61)	34.0 (21.5–67.0)	31.0 (19.0–56.0)	0.529
Alanine aminotransferase (IU/L)	26.0 (16–49)	27.0 (16.5–60.5)	27.0 (15.8–40.3)	0.477
Lactate dehydrogenase (IU/L)	252.0 (199–360)	249.0 (208–310)	285.0 (171–492)	0.688
Blood urea nitrogen (mg/dL)	15.1 (10.6–23.2)	16.8 (11.4–24.9)	13.3 (10.1–22.0)	0.044
Creatinine (mg/dL)	0.79 (0.61–1.04)	0.81 (0.64–1.17)	0.73 (0.58–0.94)	0.048
C-reactive protein (mg/dL)	9.8 (3.9–17.4)	10.4 (5.0–17.6)	9.6 (3.4–16.9)	0.779
D-dimmer (µg/mL)	3.2 (1.93–8.32)	3.7 (2.06–8.56)	2.9 (1.78–8.33)	0.619
FDP (µg/mL)	9.5 (6.3–27.6)	9.6 (6.1–27.6)	8.7 (6.4–29.3)	0.745
PT-INR	1.2 (1.08–1.24)	1.1 (1.06–1.24)	1.2 (1.08–1.23)	0.460
Sodium (mEq/L)	136.0 (132–139)	136.0 (133–139)	136.0 (132–138)	0.833
Potassium (mEq/L)	3.9 (3.6–4.4)	3.9 (3.5–4.4)	4.1 (3.6–4.4)	0.250
Chloride (mEq/L)	100.0 (97–102)	100.0 (97–102)	99.0 (97–102)	0.697
Fe (µg/dL)	16.0 (12.0–26.5)	16.0 (11.0–26.0)	16.0 (12.8–27.5)	0.909
TIBC (µg/dL)	177.0 (139.5–220.0)	179.5 (145.3–251.3)	176 (135–210)	0.204
Serum ferritin (ng/mL)	478 (257–983)	334 (191–713)	659 (361–1493)	<0.001

All variables are expressed as median (interquartile range) and were compared using the Mann–Whitney U test. FDP: fibrin/fibrinogen degradation products, PT-INR: prothrombin time-international normalized ratio, TIBC: total iron binding capacity.

**Table 3:** The results of the multivariable logistic regression analysis.

Predictors of Each Model	OR	95% CI	*p* Value
Boruta-selected model			
ln[serum ferritin (ng/mL)]	1.988	1.373–2.878	<0.001
Platelet count (×10^4^/µL)	1.060	1.031–1.091	<0.001
Neutrophil count (%)	0.999	0.998–1.001	0.521
White blood cell count (×10^3^/µL)	0.999	0.999–1.000	0.509
Lactate dehydrogenase (IU/L)	1.000	0.999–1.001	0.714
LASSO-selected model			
ln[serum ferritin (ng/mL)]	2.761	1.669–4.567	<0.001
Chloride (mEq/L)	1.276	1.076–1.513	0.005
Platelet count (×10^4^/µL)	1.083	1.042–1.125	<0.001
Age	1.012	0.986–1.039	0.374
Lactate dehydrogenase (IU/L)	1.002	0.998–1.005	0.311
White blood cell count (×10^3^/µL)	0.999	0.999–1.000	0.581
C-reactive protein (mg/dL)	0.998	0.9454–1.054	0.949
Aspartate aminotransferase (IU/L)	0.996	0.985–1.006	0.409
Alanine aminotransferase (IU/L)	0.993	0.979–1.007	0.331
Neutrophil count (%)	0.984	0.960–1.008	0.181
Blood urea nitrogen (mg/dL)	0.965	0.930–1.002	0.064
Sodium (mEq/L)	0.905	0.791–1.036	0.149
Albumin (g/dL)	0.810	0.395–1.662	0.565

**Table 4:** Stratified likelihood ratios of the prediction model in the present cohort.

Score	Likelihood Ratio	Infection	Non-Infection
−7.10 to −1.00	0.13	32	4
−0.99 to −0.16	0.74	21	15
−0.14 to 0.75	1.64	14	22
0.78 to 3.26	5.04	6	29

**Table 5:** Validation of the predictive model with the cutoff points determined in the present study.

Statistics for 3 Cutoff Points	Using Cutoff Value of the Present Study
Sensitivity 90%	
Cutoff value for scores	−0.85
Probability ^†^	29.8
Sensitivity	90.0
Specificity	50.7
Positive predictive value	36.4
Negative predictive value	84.1
Youden’s index	
Cutoff value for scores	−0.21
Probability ^†^	49.4
Sensitivity	77.1
Specificity	68.5
Positive predictive value	71.9
Negative predictive value	69.6
Specificity 90%	
Cutoff value for scores	0.53
Probability ^†^	62.9
Sensitivity	51.4
Specificity	90.4
Positive predictive value	83.3
Negative predictive value	65.3

^†^: The value was calculated as the probability of a prediction model for infectious disease among patients with undiagnosed fever.

## Data Availability

The datasets generated during the current study are registered in the University Hospital Medical Information Network at www.umin.ac.jp (ID: UMIN000054580) and are available from the corresponding author on reasonable request.

## Supplementary Materials

The following supporting information can be downloaded at https://www.mdpi.com/article/10.3390/jcm15051905/s1; Figure S1: The results of candidate variables for the model identified by two machine-learning approaches; Table S1: Breakdown of causative diseases in the infectious disease and non-infectious disease groups; Table S2: Execution rate of each examination to identify the cause of fever; Table S3: Platelet counts, serum ferritin levels, urea nitrogen and creatinine levels for each causative disease.

## Abbreviations

The following abbreviations are used in this manuscript:

AUC	Area under the curve
BIC	Bayesian Information Criterion
CI	Confidence interval
CT	Computed tomography
ICD-10	International Statistical Classification of Diseases and Related Health Problems-10th Revision
IQR	Interquartile range
LASSO	Least Absolute Shrinkage and Selection Operator
LDH	Lactate dehydrogenase
MRI	Magnetic resonance imaging
NIID	Noninfectious inflammatory diseases
NPV	Negative predictive value
OR	Odds ratio
PPV	Positive predictive value
SF	Serum ferritin
