# Supplementary material for: Development of a Machine Learning-Based Prediction Model to Differentiate Infectious and Non-Infectious Diseases in Patients with Undiagnosed Fever: A Single Hospital-Based Retrospective Study
Source: J Clin Med. 2026 Mar 2;15(5):1905. doi: 10.3390/jcm15051905 (PMC12986460; doi:10.3390/jcm15051905)

Supplement Table S1: Breakdown of causative diseases in the infectious disease and non-infectious disease groups

Group	Disease group	Name of disease in detail
infectious disease group (N=73)	bacterial infection (N=50)	Pneumonia(5), Infective endocarditis(3), Erythema japanense(3), Urinary tract infection(3), Bacteremia(3), Pyogenic spondylitis(2), Infective aneurysm(2), Acute prostatitis(2), Pyelonephritis(2), Infectious hepatic cyst (1), Bacterial meningitis (1), Salmonella enteritis (1), AFBN (1), Renal abscess (1), Central line infection (1), Colonic diverticulitis (1), Epidural abscess (1), Perianal abscess (1), Bacterial Enteritis (1), Artificial Joint Infection (1), Septic Shock (1), Febrile Neutropenia (1), Sinusitis (1), Rickettsial Infection (1), Listeria Infection (1), Fallopian Tube Abscess (1), Pharyngotonsillitis (1), Necrotising fasciitis (1), Pyogenic arthritis (1), Acute cholecystitis (1), Sternal osteomyelitis (1), Brain abscess (1), Acute cholangitis (1), Hip abscess (1), Dialysis shunt infection (1)
	viral infection (N=22)	Viral infections (14), aseptic meningitis (3), SFTS (2)<br>EBV reactivation (1), viral myocarditis (1), cytomegalovirus hepatitis (1)
	acid fast infection (N=1)	pulmonary tuberculosis (1)
non-infectious disease group (N=70)	non-infectious inflammatory disease (N=43)	Adult Still's disease (7), Rheumatoid arthritis (3), Wegener's granulomatosis (3), Polymyalgia rheumatica (2), Crystalloid arthritis (2), SLE (2), Idiopathic hypereosinophilic syndrome (2), AL amyloidosis (1), Ulcerative colitis (1), Malignant rheumatoid arthritis (1), Pseudogout (1), Thyrotoxicosis (1), Sarcoidosis (1), Sjögren's syndrome (1), Periodic fever syndrome (1), Reactive arthritis (1), Dermatomyositis (1), Incomplete Mediterranean Fever (1), Summer Type Hypersensitivity Pneumonitis (1), Polyarteritis nodosa (1), Serum Reaction Negative Spondyloarthropathy (1), Microscopic Polyangiitis (1), Anti-ARS Antibody Syndrome(1), Histiocytic Necrotizing Lymphadenitis(1), Femoral Myositis(1), Aortitis Syndrome(1), Mesenteric Lymphadenitis(1), Reactive Arthritis(1), incomplete type of Behçet's Disease(1)
	malignant tumor (N=15)	Malignant lymphoma (3), Diffuse large B-cell lymphoma (2), Gastric cancer (1), Paraneoplastic syndrome (1), Urothelial carcinoma (1), Burkitt's lymphoma (1), EBV positive diffuse large B-cell lymphoma (1) Adult T-cell leukaemia lymphoma (1), Peripheral T-cell lymphoma (1), Peritoneal carcinoma (1), Hodgkin lymphoma (1), Gastrointestinal stromal tumour of the ileum (1)
	other disease (N=12)	Malignant syndromes (3), Drug eruptions (1), Hepatic hemangioma haemorrhagic fever (1), VAHS (3), Non-infectious encephalopathy (1), Pulmonary embolism (1), Drug-induced pancreatitis (1), Myositis of unknown origin (1)

AFBN: Acute focal bacterial nephritis, ARS: Aminoacyl tRNA synthetase, AL: Amyloid light chain, EBV: Epstein-Barr virus, SFTS: Severe fever with thrombocytopenia syndrome, SLE: Systemic lupus erythematosus, VAHS: Virus-associated hemophagocytic syndrome.

Supplement Table S2: Execution rate of each examination to identify the cause of fever.

	Infection group (N = 73)	Non-infection group Total (N = 70)	NIID† (N = 43)	Malignancy (N = 15)	Others (N = 12)
Laboratory Findings					
Blood test	73 (100)	70 (100)	43 (100)	15 (100)	12 (100)
IGRA in tuberculosis	23(32)	35(50)	20(47)	8(53)	7(58)
RPR/TPHA	33(45)	48(69)	30(70)	9(60)	9(75)
Beta-D-glucan	15(21)	25(36)	13(30)	7(47)	5(42)
Urinary test	73 (100)	70 (100)	43 (100)	15 (100)	12 (100)
Cerebrospinal fluid test	15 (21)	6 (9)	4 (9)	NA	2 (17)
Cultures					
Blood culture	73 (100)	67 (96)	40 (93)	15 (100)	12 (100)
Urine culture	37(51)	35(50)	16(37)	13(87)	6(50)
Sputum culture	18(25)	17(24)	7(16)	6(40)	4(30)
Sputum acid-fast bacilli culture	8(11)	11(16)	6(14)	5(33)	0(0)
Cerebrospinal fluid culture	15(21)	6(9)	3(7)	NA	3(25)
Echocardiography	45 (62)	53 (76)	34 (79)	8 (53)	11 (92)
Transthoracic echocardiography	45 (62)	53 (76)	34 (79)	8 (53)	11 (92)
Transesophageal echocardiography	6 (8)	NA	NA	NA	NA
Endoscopy					
Esophagogastroduodenoscopy	19 (26)	31 (44)	19 (44)	8 (53)	4 (33)
Total Colonoscopy	3 (4)	21 (30)	16 (37)	3 (20)	2 (17)
Radiological Findings					
Chest X-ray	73 (100)	70 (100)	43 (100)	15 (100)	12 (100)
Cranial computed tomography	20 (27)	15 (21)	8 (19)	2 (13)	5 (42)
Thoracic computed tomography†	45 (62)	57 (81)	37 (86)	11 (73)	9 (75)
Cranial magnetic resonance imaging	23 (32)	12 (17)	9 (21)	1 (7)	2 (17)
Spinal magnetic resonance imaging	7 (10)	5 (7)	5 (12)	NA	NA
Other examinations					
Cytology	12(16)	12(17)	4(9)	6(40)	2(17)
Pathological examination	20 (27)	49 (70)	29 (67)	12 (80)	8 (67)

All values are n (%). IGRA: interferon-gamma release assay, NA: not available, NIID: non-infectious inflammatory disease, TPHA: treponema passive hemagglutination, RPR: rapid plasma regain.

†: with contrast enhancement

Supplement Table S3: Platelet counts, serum ferritin levels, urea nitrogen and creatinine levels for each causative disease

	Infection (N=73)	NIID(N=43)	Malignancy (N=15)	Others (N=12)
PLT ($\times 10^4/\mu L$)	18.8(10.9-26.5)	36.2(22.1-45.5)	15.1(7.1-32.6)	17.5(14.9-27.7)
SF (ng/mL)	334(191-713)	515(335-962)	1020(773-2652)	949(301-8081)
BUN (mg/dL)	16.8(11.4-24.9)	11.5(9.8-16.5)	18.7(11.8-25.1)	16.2(12.2-30.7)
Creatinine (mg/dL)	0.81(0.64-1.17)	0.68(0.56-0.86)	0.75(0.60-0.99)	0.96(0.71-1.39)

BUN: blood urea nitrogen, NIID: non-infectious inflammatory disease, PLT: platelet count, SF: serum ferritin,

Supplement Figure S1: The results of candidate variables for the model identified by two machine-learning approaches

a) Boruta algorithm

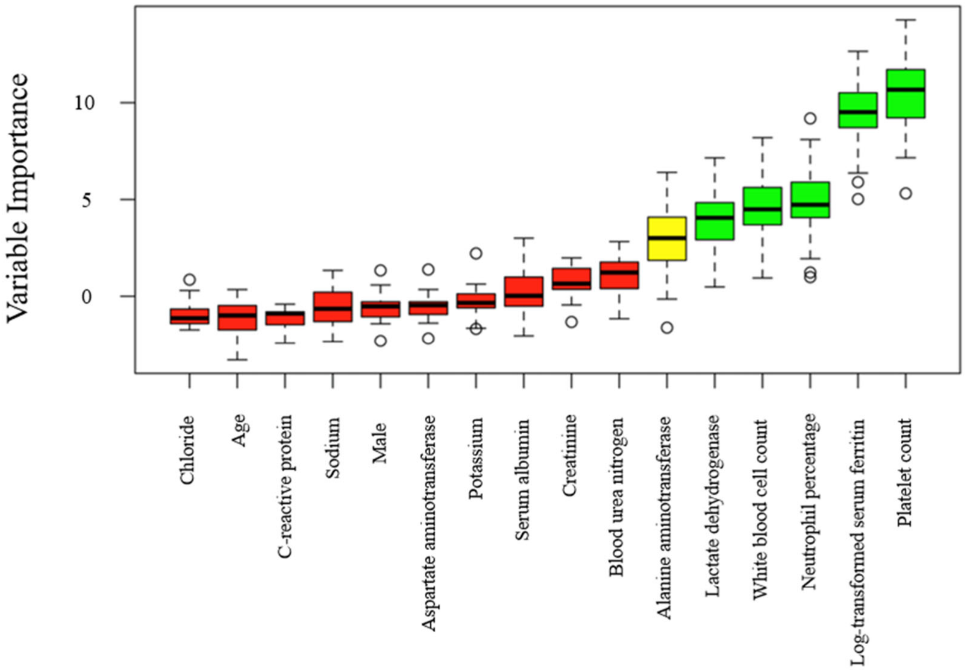

b) Least Absolute Shrinkage and Selection Operator (LASSO) regression

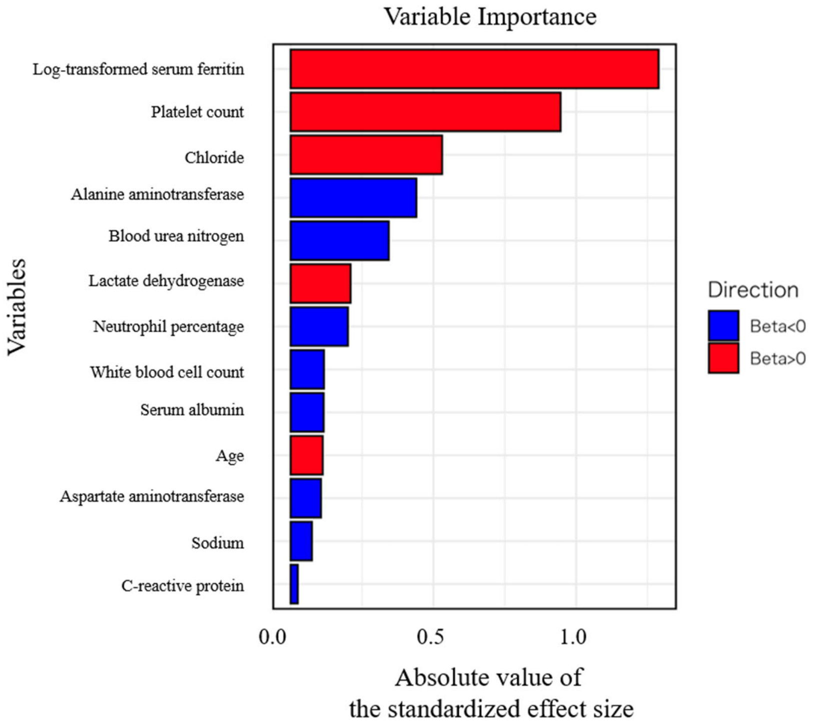

Supplement: Supplementary file 1 [file jcm-15-01905-s001.zip › jcm-4123309-supplementary.pdf]
